# Hidden blood loss and its influential factors after total hip arthroplasty

**DOI:** 10.1186/s13018-015-0185-9

**Published:** 2015-03-18

**Authors:** Kaisong Miao, Su Ni, Xianju Zhou, Nanwei Xu, Rongbin Sun, Chao Zhuang, Yuji Wang

**Affiliations:** Department of Orthopaedics, Changzhou No.2 People’s Hospital, the Affiliated Hospital of Nanjing Medical University, 29 Xinglong Alley, Changzhou, 213003 People’s Republic of China; Laboratory of Clinical Orthopaedics, Department of Orthopaedics, Changzhou No.2 People’s Hospital, the Affiliated Hospital of Nanjing Medical University, 29 Xinglong Alley, Changzhou, 213003 People’s Republic of China; Laboratory of Neurological Diseases, Department of Neurology, Changzhou No.2 People’s Hospital, the Affiliated Hospital of Nanjing Medical University, 29 Xinglong Alley, Changzhou, 213003 People’s Republic of China

**Keywords:** Hidden blood loss, Influential factors, Total hip arthroplasty, Gross formula, Multiple and stepwise regression

## Abstract

**Background:**

Total hip arthroplasty (THA) is a vital therapy for various hip joint diseases. However, patients have lower hemoglobin level post-operatively, remarkably inconsistent with the measured blood loss. The inconsistence is majorly attributed to hidden blood loss (HBL). In this study, we investigated the HBL and its influential factors among patients after THA.

**Methods:**

From January 2008 to June 2014, 322 patients (99 males and 223 females) undergoing THA were enrolled in this study. All patients were assessed comprehensively before the operation. The demographic information of the patients was collected. Intra-operative and post-operative blood loss was recorded, and then, the total perioperative blood loss and the HBL were calculated. Influential factors were further analyzed by multiple and stepwise regression.

**Results:**

The HBL was 429 ± 223 mL, with a percentage of 35.4% ± 11.0% in the total perioperative blood loss (1,155 ± 377 mL). Multiple and stepwise regression analysis revealed that HBL was positively associated with body mass index (BMI), blood transfusion volume, length of incision, change of hematocrit (HCT) between pre-operation and post-operation but negatively associated with age. As compared to male patients, female patients had a risk of increased HBL. Development displasia hip (DDH) patients had a less risk of HBL in all patients.

**Conclusion:**

HBL is a significant portion of total blood loss in the patients after THA. Gender, age, BMI, blood transfusion, length of incision, change of HCT, and diagnosis are influential factors of HBL.

## Introduction

Total hip arthroplasty (THA) is one of the most important treatments for many hip joint diseases, such as femoral neck fracture (FNF), osteoarthritis (OA), rheumatoid arthritis (RA), development displasia hip (DDH), and avascular necrosis of femoral head (ANFH) [1]. It is also a procedure that frequently leads to massive blood loss. Previous studies showed that the total blood loss was in the range of 1,023–1,785 mL [2-5] and 30%–69% of patients needed blood transfusion after THA [6,7]. Keating and Meding reported that transfusion is an independent risk factor for post-operative complications, such as immune disorder and infection [8].

In usual clinical practice, the blood loss measured after THA includes merely the intra-operative blood loss and the post-operative drainage. In spite of an apparently satisfactory blood management on blood loss, patients still have anemia. Liu et al. speculated that some potential factors are not considered for anemia after THA, for example, blood penetration into the tissues, blood accumulation around the joint, as well as hemolysis [4].

The potential blood loss, called hidden blood loss (HBL), is usually ignored. Until 2000, the concept of HBL was formally put forward [9]. Several theories were proposed to address it. Pattison et al. and Faris et al. initially suggested that HBL may be attributable to hemolysis [10,11]. But Erskine et al. proposed that the unexplained loss went into tissue compartments by using labeled red cells [12]. Recently, Bao et al. concluded that free fatty acids generated from fatty emboli in blood circulation are responsible for the HBL by peroxidation damage of membrane molecules of RBC and hemoglobin [13]. Moreover, there is evidence that some factors, like gender [14,15], diagnosis [4], body mass index (BMI) [16], anticoagulants [3,17], tranexamic acid [18,19], and even closed-suction drainage [20], may influence HBL. However, the mechanisms and influential factors for HBL following THA are not fully clear, and some of them are still controversial. In our study, we investigated the HBL and analyzed the influential factors of HBL after THA by using multiple and stepwise regression.

## Materials and methods

### Patients

A total of 322 patients (including 99 males and 223 females) with a complete history of disease and an average age of 71 (35–94) years were recruited in this study. The diagnosis of patients included FNF (65.5%), OA (8.1%), ANFH (10.9%), RA (7.1%), and DDH (8.4%). All the patients underwent primary THA surgery which was performed by one same orthopedic surgeon at Changzhou No.2 People’s Hospital of Nanjing Medical University from January 2008 to June 2014. Patients who underwent previous operations on the hip were excluded. All enrolled patients had no history of hematological diseases which could affect severely blood coagulation. Variables such as gender, age, height, weight, BMI, length of incision, pre-operative and post-operative hematocrit (HCT), intra-operative blood loss, post-operative drain blood volume, transfusion blood volume, and reinfusion volume of drained blood were recorded.

This study was reviewed and approved by the Ethics Committee of Changzhou No.2 People’s Hospital. And informed consent was received from all patients.

### Intra-operative treatment

The surgery was performed under general anesthesia or continuous epidural anesthesia. All the patients received a standard operating procedure via the posterior-lateral incision. Length of incision was within a range of 10 to 15 cm. The prosthetic types of implants were divided into cemented and uncemented. The intra-operative blood loss was calculated by weighing the used compresses and recording the amount of blood in the suction bottle and the filtrated drainage blood which was recycled and transfused to patients by self-blood transfusion equipment during operation.

### Post-operative treatment

The drainage tube was removed 48 h after operation, and the drainage volume was recorded as the post-operative blood loss. During the post-operative period, hemostatic such as tranexamic acid was not used and low molecular weight heparin (LMWH) was routinely injected to prevent deep venous thrombosis. Qiong Danshen injection triazine (a kind of Chinese medicine) or cinepazide was used to improve microcirculation and facilitate recovery of patients. After removal of drainage tube, patients were requested to take an x-ray in order to examine the location of the prosthesis and encouraged to walk with a walking aid.

### Calculation of HBL

The patient’s blood volume (PBV) was calculated by Gross formula: PBV = k1× h^3^ + k2 × w + k3, (h, height (m); w, weight (kg); for male, k1 = 0.3669, k2 = 0.03219, and k3 = 0.6041; for female k1 = 0.3561, k2 = 0.03308, and k3 = 0.1833) [21]. The total perioperative blood loss was calculated by multiplying PBV by the change of HCT, and HBL was further calculated. The perioperative blood loss was the sum of intra-operative blood loss, post-operative drainage volume, transfusion volume, and HBL.

### Statistical analysis

Multiple and stepwise regression analysis was performed to evaluate the influential factors of HBL using fourteen independent variables, including seven qualitative variables (age, BMI, PT, blood transfusion volume, operation time, length of incision, and change of HCT between pre-operation and post-operation) and seven quantitative variables (sex, hypertension, anesthesia method, prosthetic type, anticoagulants, hemorheologic agent, and diagnosis). In the quantitative variables, male, hypertension, general anesthesia, uncemented prosthesis, nonuse of LMWH, nonuse of hemorheologic agent, and FNF diagnosis were set as “0”, others were converted into dummy variables. A positive coefficient indicates a positive influence on the dependent variable (HBL), whereas a negative coefficient indicates a negative influence. All independent variables were incorporated into the model using the method of “Enter”. All statistical analyses were performed by SPSS for Windows Ver.18.0 (SPSS Inc. IL, USA), and differences at a level of *P* < 0.05 were identified statistically significant.

## Results

Between January 2008 and June 2014, a total of 322 patients underwent unilateral primary THA were enrolled consecutively in this study. Demographic data of patients are summarized in Table 1. There are no differences in age, BMI, and hospital stay between men and women (*P* > 0.05, *χ*^2^ test). The data for operation time, length of incision, intra-operative blood loss, wound drainage, re-infused blood volume, hematocrit level loss, calculated blood loss, HBL, total blood loss, and the percentage of HBL are shown in Table 2. The mean total blood loss was 1,155 ± 377 mL, and the HBL was 429 ± 223 mL (35.4% ± 11.0% in total blood loss), indicating a considerable amount of HBL.

**Table 1:** **Patient’s demographic information**

**Parameters**	**Male**	**Female**	**Total**
Number of patients	99	223	322
Age (years)*	68 (35–92)	72 (44–94)	71 (35–94)
Height (m)	1.69 (0.05)	1.57 (0.05)	1.61 (0.07)
Weight (kg)	70.6 (10.3)	58.3 (9.2)	62.1 (11.1)
BMI (kg/m^2^)	24.6 (3.3)	23.5 (3.4)	23.8 (3.4)
Hospital stay (days)*	17 (9–36)	17 (8–37)	17 (8–37)

Other values are presented as mean (SD).

*SD* standard deviation.

*Values are presented as median.

**Table 2:** **Perioperative blood changed in the patients after THA**

**Parameters**	**Values**
Number of patients	322
Operation time (min)	88 (26)
Length of incision (cm)	13.4 (1.6)
Intra-operative blood loss (mL)	411 (143)
Wound drainage (mL)	314 (157)
Reinfused blood (mL)	604 (389)
Hematocrit level loss (%)	14.3 (4.1)
Calculated blood loss (mL)	551 (196)
Hidden blood loss (mL)	429 (223)
Total blood loss (mL)	1,155 (377)
Percentage of hidden loss in total (%)	35.4 (11.0)

Values are presented as mean (SD).

To next examine the association between HBL and fourteen influential factors as mentioned earlier, we performed multiple and stepwise regression analysis. First, we found that the regression analysis is reliable (its imitation degree is ideal, *R* = 0.941) and predictive (*P* < 0.001). As shown in Table 3, the following influential factors were positively correlated with HBL: BMI (*P* = 0.020), blood transfusion volume (*P* < 0.001), length of incision (*P* < 0.001), and change of HCT (*P* < 0.001). In contrast, age was negatively associated with HBL (*P* = 0.012). As compared to male patients, female patients had a risk of increased HBL (*P* = 0.033). DDH patients had a less risk of HBL in all patients (*P* = 0.030). However, it appeared that prothrombin time, operation time, hypertension, anesthesia method, prosthetic type, and use of LMWH or hemorheologic agents were not significantly correlated with HBL.

**Table 3:** **Multiple linear regression analysis on influential factors of HBL after THA**

**Variables**	**Coefficients**	***P*** **values**
Constant	−807.9	0.000*
Quantitative variable		
Age (y)	−1.2	0.012*
BMI (kg/m^2^)	3.2	0.020*
PT (s)	−0.4	0.881
Blood transfusion (mL)	0.3	0.000*
Operation time (m)	−0.1	0.613
Length of incision (cm)	69.9	0.000*
Change of HCT	11.6	0.000*
Qualitative variables		
Gender	22.4	0.033*
Hypertension	−10.8	0.247
Anesthesia method	−9.5	0.336
Prosthetic type	16.5	0.182
Anticoagulants		
LMWH (2,050 iu/day)	28.6	0.165
LMWH (4,100 iu/day)	−15.2	0.291
Hemorheologic agent		
Qiong Danshen injection triazine	−5.9	0.552
Cinepazide	−5.2	0.772
Diagnosis		
Hip osteoarthritis	−3.8	0.827
ANFH	−16.9	0.191
RA	−66.2	0.241
DDH	−38.2	0.030*

*BMI* body mass index, *PT* prothrombin time, *HCT* hematocrit, *LMWH* low molecular weight heparin, *ANFH* avascular necrosis of femoral head, *RA* rheumatoid arthritis, *DDH* development displasia hip.

*P < 0.05.

## Discussion

Although there are some reports on HBL after arthroplasty surgery, mostly focusing on total knee arthroplasty (TKA) but much less on THA, a systematic investigation on HBL after THA and its influential factors is lacking. In this study, we performed a detailed retrospective investigation on 322 consecutively enrolled THA patients with relatively complete clinical data and a relative reliable approach for estimating HBL and further comprehensively analyzed the influential factors of the HBL by the multiple regression analysis.

Emerging data suggest that there is a significant amount of HBL after THA, which is usually ignored in the clinical practice for estimating perioperative blood loss. For example, Sehat et al. reported that the mean HBL after THA was 471 mL, being 26% of the total blood loss [22]. Subsequently, using the same method of calculating PBV, Liu et al. showed that the HBL in patients with FNF was 967 mL [4]. Similarly, in our study, the volume of HBL was 429 ± 223 mL, with a percentage of 35.4% ± 11.0% in total blood loss, indicating a considerable amount. Previous studies provided some possible explanations for the mechanisms of HBL. It was proposed that the post-operative blood loss may be, at least in part, arise from hemolysis [10]. Moreover, Faris et al. further observed the increased levels of hemolysis by using unwashed, filtered, and re-infused blood [11]. Consistently, our result showed that blood transfusion can increase the HBL after THA, which is probably due to hemolysis. Another explanation is that the HBL is presumably ascribed to blood infiltration into tissue compartments [12]. In our study, the length of incision contributed to the HBL, possibly by blood infiltration. Namely, the longer the incision was, the more penetrable tissue compartments forms. As a result, the blood would be accumulated in these compartments, ultimately leading to an increase in HBL.

Regarding influential factors for HBL, there are some reports. For instance, Shen et al. reported that gender and blood transfusion do not affect HBL in the patients after TKA, but the use of tourniquet can reduce the HBL [15]. However, Cushner and Friedman found that gender plays a role in HBL, with greater contribution in men relative to women. And they concluded that age, diagnosis, operative time, and tourniquet time are not associated with blood loss [14]. Additionally, there is evidence that the amount of HBL is also associated with diagnosis [4], BMI [16], and LMWH [17]. Our results showed that gender, age, BMI, blood transfusion, length of incision, change of HCT, and diagnosis are correlated with the HBL after THA. Further, gender, length of incision, change of HCT, and diagnosis may have stronger influences. Notably, the calculation method can also affect the result of HBL. The Gross formula is a linear model for circulating PBV by using the perioperative hematocrit. It was initially introduced by Ward and then improved by Gross [21]. Post-operative hematocrit is one of the most important reference indexes to calculate the HBL. Some researchers suggested the hematocrit value 2–3 days after operation as an index [9,22]. Based on our daily pre-operative and post-operative blood routine tests, we found that the lowest hematocrit value did not occur on the second or third day after surgery in most patients but on the fourth to seventh day. In agreement with our result, it was reported that the hemoglobin (HB) level of most patients initially declines and then increases on the seventh day after arthroplasty surgery [15,23]. In other words, the calculated PBV would be lower than the actual volume if the hematocrit value is used as a reference 2–3 days after surgery, leading to underestimate the HBL. Thus, we used the hematocrit value 4–7 days after operation to calculate the HBL.

In this study, we could not rule out the influence of racial differences on our results because our subjects were from the local native population. Additionally, we did not use tranexamic acid, a common drug for hemostasis which is routinely used in many surgeries. Recently, it was reported that it can reduce post-operative blood loss and transfusion rate after THA [24-26]. But as an antifibrinolytic, it had a potential thrombotic effect; thus, it was contradicted in those patients at risk or with a history of thrombosis [27]. In our hospital, the drug was not applied routinely during the perioperative period and as a result possibly leading to the increase of the perioperative blood loss. Finally, other potential influential factors, such as the cup size of implant and bone mineral density of patients, which were not included in this study, need to be further determined.

In conclusion, HBL is a significant portion of total blood loss in the patients after THA. Gender, age, BMI, blood transfusion, length of incision change of HCT, and diagnosis are influential factors of HBL in patients after THA. However, the HBL appears not to be associated with hypertension, anesthesia method, prosthetic type, pre-operative prothrombin time, operation time, dosage of LMWH post-operative, and use of blood-activating drug post-operative. Lowest post-operative HCT should be used for calculating the HBL. By focusing on these factors, further clinical research should be done to reduce the HBL. If HBL can be successfully controlled after surgery, the rehabilitation of patients will be improved after THA.
